# Supplementary material for: Persistent Transmission of HCV among Men Who Have Sex with Men despite Widespread Screening and Treatment with Direct-Acting Antivirals
Source: Viruses. 2022 Sep 2;14(9):1953. doi: 10.3390/v14091953 (PMC9502248; doi:10.3390/v14091953)
Supplement: Supplementary file 1 [file viruses-14-01953-s001.zip › viruses-1869501-supplementary.pdf]

Supplemental information

Table S1

	All patients (n=80)	HCV genotype 1a (n=51)	HCV genotype 4 (n=29)
Age (years)	47 (10)	47 (10)	49 (8)
Male sex	80 (100%)	51 (100%)	29 (100%)
Race			
White	72 (90%)	46 (90%)	26 (90%)
HCV transmission route			
MSM	80 (100%)	51 (100%)	29 (100%)
Current episode is a reinfection	19 (24%)	12 (24%)	7 (24%)
Number of HCV episodes			
1	61 (76%)	39 (76%)	22 (76%)
2	15 (19%)	9 (18%)	6 (21%)
≥3	2 (3%)	3 (6%)	1 (3%)
Alanine aminotransferase concentration (IU/mL)	139 (74–315)	144 (76–310)	136 (70–427)
HCV RNA concentration (IU/mL)	310 000 (34 000–1 400 000)	330 000 (31 000–1 400 000)	250 000 (49 000–1 500 000)
Time between estimated infection date and HCV treatment (months)	4·4 (1·2)	4·3 (1·1)	4·6 (1·3)
Time between first positive HCV RNA test and HCV treatment (months)	2·0 (1·0)	2·0 (1·0)	2·0 (1·1)
HBV co-infection	0	0	0
HIV co-infection	73 (91%)	46 (90%)	27 (93%)
CD4 cell count (per μL)	605 (490–765)	601 (497–770)	610 (396–767)
HIV viral load <50 copies per mL	71 (97%)	45 (98%)	26 (96%)
Patient on combination ART	73 (100%)	46 (100%)	27 (100%)

Data are mean (SD), n (%), or median (IQR). HCV=hepatitis C virus. MSM=men who have sex with men. HBV=hepatitis B virus. ART=antiretroviral therapy.

***Table 1:* Baseline characteristics**

Baseline characteristics	DAHHS (n=57)
Sex, male	57 (100%)
Age, years, median (IQR)	40 (34-47)
CD4 count, E9/L, median (IQR)	0·66 (0·45-0·79)
ALT, U/L, median (IQR)	160 (67-416)
Bilirubin. umol/L, median (IQR)	10 (6·5-13)
HCV genotype 1	
subtype a	54 (95%)
subtype b	3 (5%)
unknown	0
HCV RNA IU/mL, median (IQR)	222.000 1.235.000)
Treatment duration, weeks, median (IQR)	12 (12-12)
Interval infection-treatment, weeks, median (IQR)	22 (16·5-25)
Il28B genotype	
RS 1297, CC	25 (42·5%)
RS 1297, non-CC	30 (57·5%)

Table S2 – Phylogenetic comparison of whole genome sequences versus concatenated NS5A+NS5B sequences of

the DAHHS 1 samples

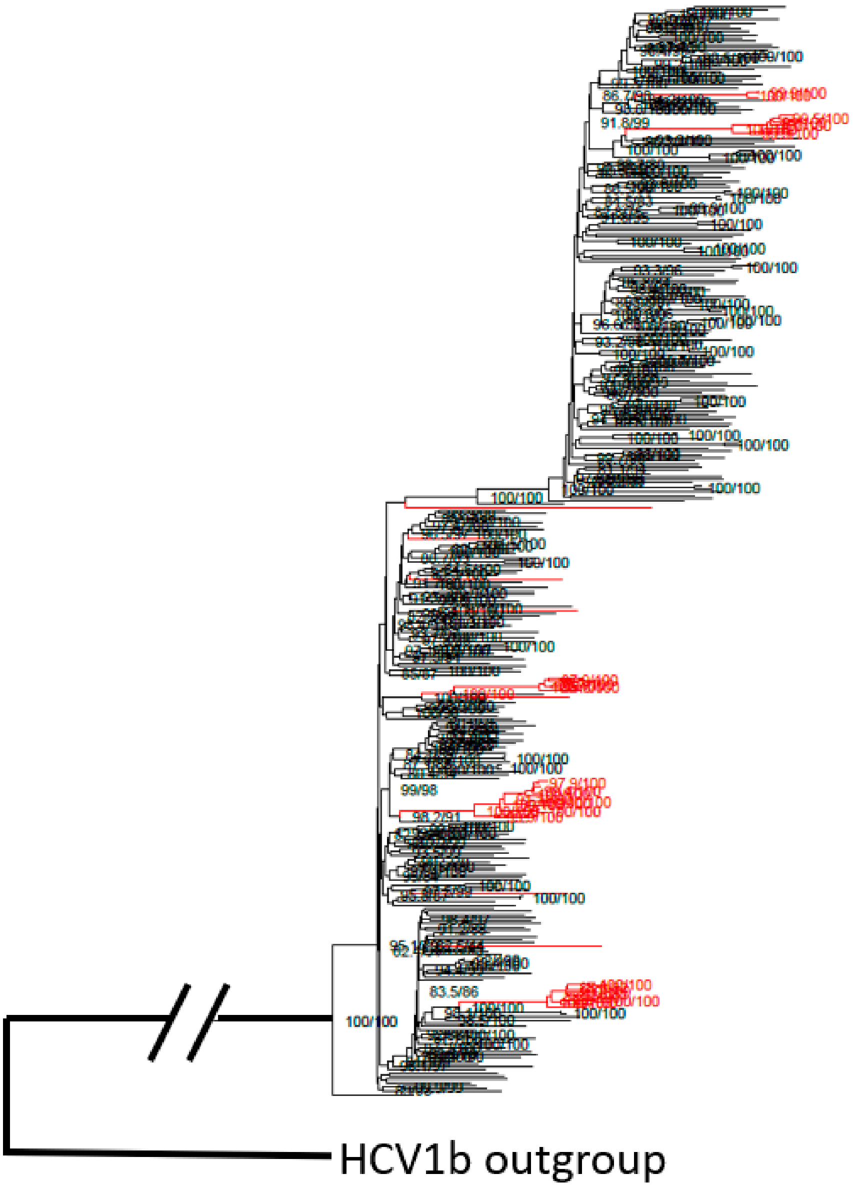

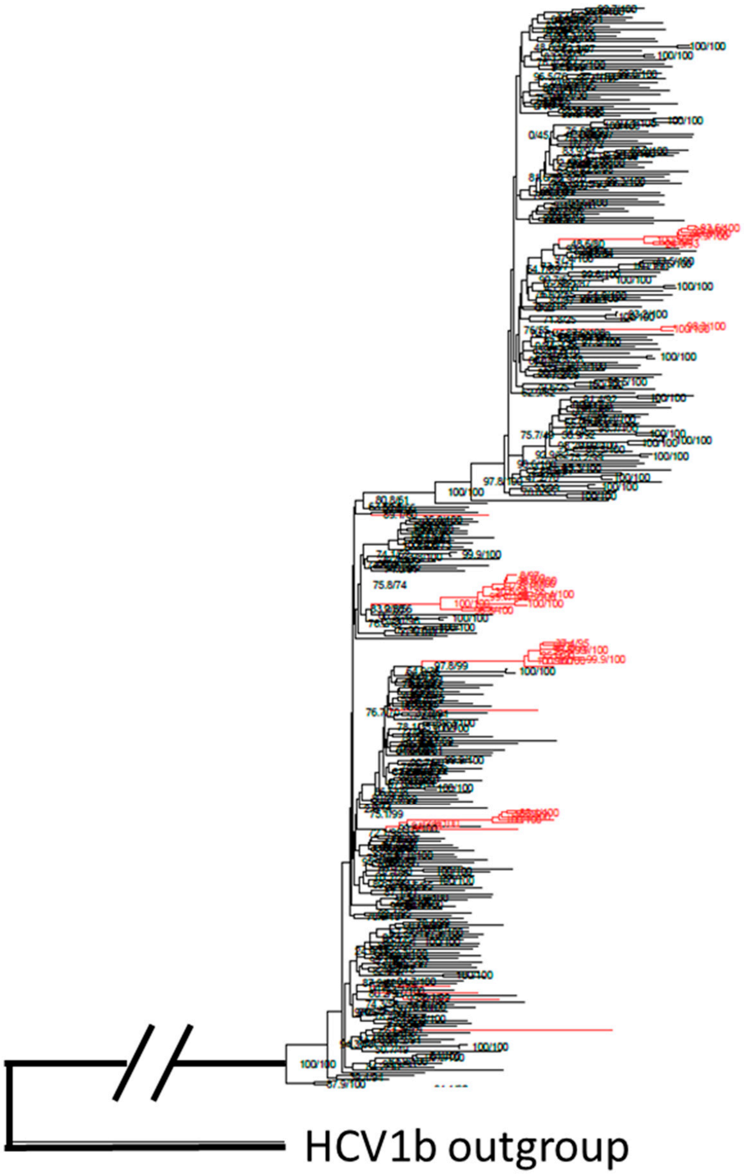

Figure S1: Upper phylogenetic tree consists of the whole genome sequences of the DAHHS 1 samples. The lower phylogenetic tree consists of the same DAHHS 1 samples only we used the concatenated NS5A+NS5B parts. Maximum likelihood analysis was performed with a GTR+G4+I model. We did not find any differences in the cluster structure and number of clusters in the tree.
